# Commentary: The Concept of a Bewusstseinskultur

**DOI:** 10.3389/fpsyg.2018.00732

**Published:** 2018-07-30

**Authors:** Sascha Benjamin Fink

**Affiliations:** ^1^Program for Philosophy-Neurosciences-Cognition, Institute III: Philosophy, Otto-von-Guericke-University Magdeburg, Magdeburg, Germany; ^2^Center for Behavioral Brain Sciences, Magdeburg, Germany; ^3^DFG Research Training Group 2386 “Extrospection”, Humboldt-Universität zu Berlin, Charité Berlin, Otto-von-Guericke-Universität Magdeburg, Max Planck Institute for Human Cognitive and Brain Sciences, Leipzig, Germany

**Keywords:** Bewusstseinskultur, transformative experiences, consciousness culture, phenomenal value, phenomenal consciousness, subjective value, functionality of consciousness, consciousness ethics

## 1. Introduction

Thomas Metzinger has diagnosed the need for a *Bewusstseinskultur*,^1^ a ‘consciousness culture’: a culturally implemented way in which a society as a whole engages with the dawning natural science of consciousness, with phenomenal experiences themselves, and with our increasing capability to manipulate them. A Bewusstseinskultur is an achievement, built by a society-wide orientation on empirical evidence, thorough scientific theorizing and rational deliberation. It affects a broad range of issues from animal ethics, drug policy, end-of-life-care, and robo-ethics to post-humanism.^2^

The goal of Bewusstseinskultur is not academic, but practical:^3^ to change our society for the better, based on our best philosophical and empirical view of who we are, how we work, and what we can achieve. Unfortunately, most of Metzinger's writings on this issue have been in popular media—and in German (Metzinger, 1994, 1998, 2000a,b, 2005, 2006c, 2016, 2017a).^4^ But the time is ripe to bring this visionary concept onto the international stage and emphasize its academic merits. So, what is Bewusstseinskultur and what hurdles lie on the way to attain it?

## 2. What is bewusstseinskultur?

Metzinger (2006a, p. 15) mainly defines Bewusstseinskultur by what it leads to: (i) a rational way of implementing new scientific discoveries and new ways of acting, of manipulating ourselves and our environment, and (ii) a timely embedding of scientific, theoretical, and technological developments into a cultural evolution. It thereby combines three parts.

First, at its core is a *Bewusstseinsethik*—a *value assessment* of conscious experiences. Bewusstseinsethik is a specialized ethics which focuses on acts that (as their primary goal) bring about, inhibit, maintain, or change conscious states. It evaluates such acts based on assessments of the resulting conscious states' values. For example, I can give you one of two drugs with the same side effects and risks before your surgery. Drug *A* paralyzes you and inhibits the formation of consciousness; drug *B* also paralyzes you, but only inhibits the formation of any kind of memory. After surgery, you will function the same, independent of whether I gave you *A* or *B*, so there is no intersubjectively available difference. But *B* likely failed to prevent terrible experiences like pain, fear, anxiety, helplessness, dread, or panic.^5^ We can hardly shake off the intuition that giving *B* instead of *A* is condemnable. All else being equal, apparently, conscious experiences themselves carry some value, which justify evaluative judgements and normative claims. We might think that experiences have an objective value, but this might be hard to establish: Some drug highs might feel wonderful subjectively, but are objectively detrimental. Metzinger himself is not a moral realist and therefore rejects the idea that the value of an experience is objective (Metzinger, 2014, p. 347f). However, one could still hold the idea that the felt subjective value can be roughly generalized over all individuals, e.g. that anybody feels headaches as bad, and the extent of their badness as being dependent on their felt quality. If this is the case, there might still be influencing factors on an experiences overall value. Maybe the costs of attaining it are too high, or experiencing it endangers attaining other valuable stes. But, first, the values should at least be somewhat intersubjectively generalizable and knowable in order to justify society wide policies based on the values of experiences themselves, and second, these values should not be parasitic on the values of the effects these experiences have in order to deserve the label Bewusstseinsethik as an ethics focusing on conscious experiences. So: When are we allowed to cause pains? Must we show children love? Is someone allowed to have psychedelic experiences?

The second ingredient of a Bewusstseinskultur is an evaluation of the likely impact on society which consciousness-manipulating technologies will have. Such a *technology assessment* has been apparent in Metzinger's own papers on robot ethics (Metzinger, 2013a) or the ethics of virtual reality (Madary and Metzinger, 2016). Any assessment is based on our Bewusstseinsethik and it leads to recommendations about how to use and implement such technologies to further societal progress and to minimize social and moral costs.

Lastly, a Bewusstseinskultur will involve an *anthropology assessment*: Our society has an implicit view of what humans are and what we are capable of, which shows itself mainly in our practices and how they are justified—akin to what Sellars (1991) called the *manifest image*.^6^ Our legal system, for example, is inherently guilt- and punishment-centred: We can punish someone *after* a crime, but we cannot force procedures on that person that prevent certain behaviors *before* one committed a crime. Guilt in the criminal sense increases with intent: Harming a person by accident is less blameworthy than harming a person with intent. This presupposes that one's practical intention causally contribute to one's acts. If our best scientific theories suggest that intentions are ineffective or mere *post fact* rationalizations, many may see this as undermining the manifest justifications of our legal practices, leading to a factual undermining of our legal practices due to an erosion of backup in the community. But giving the scientific image its due does not directly entail that our practices have to change. We can often (but not always) provide new justifications compatible with the scientific view. We therefore need not only care about implementing emerging technologies into our societies, but also about the anthropologies we offer for adoption. Because the emerging anthropology of natural science differs from the manifest image, we should worry about the transition phase in which a broad range of people may favor one of the other. We should develop strategies on how to keep the societal costs of such shifts at a minimum. But rejecting or ignoring the naturalistic view of humankind and hope that the manifest image will simply prevail is not an option.

Bewusstseinskultur must work on all three of these fronts, and it is of utmost importance in any society eager to adopt a scientific view of itself and willing to accept in large numbers the reducibility of consciousness to physical events. However, I believe that there are distinct obstacles, which take issue with the Bewusstseinsethik's central claim that the value of some experience is generalisable, knowable, or determined. These hurdles, I believe, shape the Bewusstseinskultur we can aim for.

## 3. Hurdles on an uphill road

Let's be blunt: No society has achieved anything resembling a Bewusstseinskultur. Those societies which shaped empirical science as we currently practice it have no specific canon for cultivating experiences unless these states are coupled with a capacity for economic exploitation—the current trend in Silicon Valley to micro-dose LSD in order to increase productivity or the US Army using mindfulness techniques to improve performance and resilience in fighting scenarios are prime examples. One has these experiences in order to increase some mental capacity, not primarily for their own sake. In contrast, those cultures that care for cultivating specific experiences per se are in disrepute of being esoteric, hippy, or navel-gazing—dropping out in order to meditate for several weeks or taking a day off to enjoy a “trip” is then labelled as non-productive or a waste of time. In most society-wide debates involving consciousness—animal and robot ethics, drug policy, thanatology, pedagogy, media anthropology, etc.—empirical evidences about how our mind is dependent on the minute mechanistic workings of our bodies are generally ignored. Instead, public discourse is rather shaped by ignorance, ideologies, or inconsistencies—concerning animal pain, for example, we reject the idea that animals feel pain in order to justifiably hold them in confined spaces and kill them for consumption but simultaneously presume that they can feel pain in order to perform experiments concerning the effectivity of analgesics (see Rollin, 1989, 113–118).

This is merely the state of affairs on which we could improve. However, there are some philosophical hurdles on the road toward a Bewusstseinskultur.

A Bewusstseinskultur is dependent on ascribing specific values (or, at least, value ranges) to types of experiences, e.g., pains feel bad. One could argue that the value of an experience is simply due to our linguistic practices. But one need not accept this. It could be that they are attached to the experiences, carrying their value independent of our linguistic or societal practices. I take the second view as more plausible, because for some novel (e.g., technologically induced) experiences, we lack any practice on how to conceptualize them, and they can still be of positive or negative value to the experiencing subject, being liked or disliked. Metzinger stresses the point repeatedly (Metzinger, 2000a, p. 64, Metzinger, 2006a, p. 6) that for some acts that affect consciousness, we lack any kind of moral intuition or guidance; and we lack a comprehensive scheme for which conscious states we want to keep and which to ban in our culture (e.g., Metzinger, 2005, p. 53)—despite the intuition that there are some obvious candidates on either side, like pain or love.^7^

According to Metzinger these values are associated with the phenomenal experiences themselves. Additionally, experiences carry *specific* values. Because only then could we come up with general recommendations on whether we should take steps toward promoting or prohibiting the occurrence of specific experiences in our culture, e.g., prevent someone from feeling pain, regret, a loss of bodily boundaries, etc. So even if we want to avoid moral realism, there is the presupposition that any type of phenomenal experience has a specific type of value *per se*, such that this value is roughly uniform across individuals: such-and-such an experience has such-and-such a value *for everyone*. This would then justify that a society focuses on *Bewusstsein* (consciousness) in order to question or justify specific culturally engrained practices by which we configure our environments - and thereby shape our experiences which are determined by these environments.

This crucial presupposition—specific environmental con-figurations lead to specific experiences that carry specific values—can be challenged in several ways.

First, by *phenomenal variation* (Locke, 1690/2008; Hohwy, 2011; Fink, 2017): Even if they are stimulated in the same way by their environment, individuals may still have different experiences. Such differences might be slight or massive, but even if they are slight, the associated value may change. Me and my synaesthete twin may hear the same tune, but what I find pleasing, my twin hates because it elicits the nauseating taste of boogers mixed with pencil lead.^8^ As a society, however, we can only focus on recommendations for configuring our shared environment. There is then, despite our Bewusstseinskultur, still no guarantee that our recommendations for changes in the environment will foster some experiences and prevent others because there might be massive intersubjective variation, even under the same environmental circumstances.

Let us ignore this possibility and focus on the second challenge from *transformative experiences*: Even if two subjects have experiences of the same type, the values they associate with them might differ, as Paul (2014, 2015) argues. Some people love the taste of Durian, the adored stinky fruit of Thailand, while some hate it with gusto. Worse still: You cannot know *before having the experience* whether you will like Durian or not. The same holds for more impactful events, from experiencing having a child, a committed relationship, LSD-trips, virtual reality, etc. Even if we know what we will experience, we will not know beforehand whether we will like it or not. Because the experience itself is “epistemically transformative”, i.e., only by having it can you know how it feels and whether we like that feeling; second, because the experience itself may be “personally transformative”, i.e., by undergoing the experience, one's value system may change: What would have been liked before is hated after the change because one is not the same person as before. Transformative experiences raise two problems for Bewusstseinskultur: First, even if we can control the environment in such a way that we ensure that a person has a specific kind of experience, we cannot know whether this has a positive or negative value for that person. Second, because neither society nor the individual undergoing the experience knows beforehand whether having it will be good or bad, we cannot make any rational decision (in a normative way) on whether that person should or should not undergo the experience. Paul's own take is that we should change the question: Don't ask whether your want to have some experience, but whether you want to find out how it changes you. Do we think there is value in such a revelation? But here, society cannot rationally decide for us. Culture is then limited to ensuring the autonomy of the subject's decision for or against undergoing experiences, even if they might be in some way transformative.

The third challenge of *phenomenal non-hedonicity* takes issue with grounding subjective value in phenomenality. Metzinger (2017b) stresses the importance of phenomenality itself for any mental state to be morally relevant: Hedonic value is grounded in phenomenality. In contrast, Parfit (2011, p. 53ff) argues that an experience's feel—its phenomenal aspect—is not what gives us reason to like or dislike them. While experiences of pain, hunger, fear, love, orgasmic bliss, eating cauliflower, touching styrofoam all have some positive or negative value, they do not (strictly speaking) *feel* good or bad. Then, the experience is not due to its phenomenal aspects desirable or not, but due to us liking or disliking them—a hedonic stance we take toward them. Consider one person's love for cauliflowers and another's hate for them (see also Dennett, 1993): The feels may be identical and simply the likes differ. Introspectively, we cannot detect that such hedonically-ladden experiences are composites of a feeling and a liking—our mental deep structure, even of phenomenal experiences, is opaque. What, then, makes us act in a way such that we avoid some experiences and seek others? Additional *meta-hedonic* desires, which stem nearly trivially from the hedonic ones: We simply don't like to be in states that we dislike (meta-hedonic), and because we dislike some experience (hedonic), we try to avoid it. Meta-hedonic desires can be rational (for we do have reasons for or against them), but hedonic desires are not. Instead, they are brute. Therefore, so Parfit, experiences of the same type cannot only come with different hedonic values (as Paul argues), but their phenomenal properties also give no reason to adopt one hedonic stance rather than another. Therefore, we need not care so much about the phenomenal aspects of experiences, but rather about the hedonic stances individuals have toward them. A culture focusing primarily on consciousness would then slightly miss the primary focus of moral concerns.

The last challenge comes from *phenomenal non-functionalism*,^9^ which asks: Do specific phenomenal experiences necessarily have certain effects at all? According to Paul, they do not cause *specific* likings or dislikings, and according to Parfit they do not cause (nor give reason for) likings or dislikings at all. Metzinger seems conflicted here, but it matters for the project of a Bewusstseinskultur. On the one hand, he writes that phenomenal consciousness needs to be described on a functional level (Metzinger, 2004, p. 110). On the other hand, he states that if any mental state is phenomenally conscious, it is globally available to all different subroutines (Metzinger, 2004, 17, 35, 88, 126ff, 210, 545), which is to say that it can (but need not) have effects on all (at least: a wide range) of a system's subroutines. If a mental state is globally available, we can use it for cognition, reasoning, deliberation, report, motor control and so on.

Global availability may be a prerequisite for a mental state having phenomenal aspects, but it does not differentiate types of experience because there is no individual *effect* a type of conscious state has to have in order to be exactly *this* type rather than another, e.g., an olfactory experience as of pine rather than as of sandalwood. For if a type of experience would need to have specific effects to be that experience, it could not affect all subroutines. Instead, it would only have to have causal influence on a specific set of subroutines rather than another. And its effects on subroutines could not be modal, but would need to be actual—it would not be available for, but actually affect these subroutines. For functionalism demands actual, not potential causes and effects as individuation criteria.^10^ The idea of unconstrained global availability then precludes functionalism for specific types of experience because it does not assign them specific effects. The same holds for causes in Metzinger's view: Metzinger (2004, p. 179) makes it a prerequisite of phenomenal consciousness that it can be active offline — dreams (Metzinger, 2013b), hallucinations (Metzinger, 2004, p. 173f, 365ff, 445ff), or out-of-body-experiences (Metzinger, 2009b) are examples of such states. We experience a world in some way without being in a world that matches this experience. If so, there is no individual external cause a type of experience has to have in order to be *that* type of experience rather than another. If we consider also the possibility of bringing about specific experiences with fine grained direct brain stimulation, there need not be any specific proximal or internal cause that individuates experiences. It is apparently congruent with Metzinger's self-model theory of consciousness that a type of experience is therefore not identifiable with a specific function spanning input and output of a system. Types of experiences can be statistically associated with certain functions, but cannot be identified with them because they can arise in the absence of such a function's typical causes and effects.

If experiences can be dissociated from specific causes and effects, then this sets a limit to how effective a Bewusstseinskultur can be. As a society, we can only control the *likely* causes of some experiences, but we cannot ensure that they do not arise due to *unlikely* circumstances (e.g., in dreams or while being on acid). So all our recommendations simply deal in plausibilities. Additionally, we can assume that the evaluative aspects that some experiences usually carry—pains being bad, orgasms being pleasant, and so on—are not an intrinsic part of these experiences, but are themselves the activation of some subroutine.^11^ This is best illustrated by pains, which are paradigmatically terrible experiences. But not necessarily so: Pain asymbolics are capable of experiencing pain without any evaluative component (Rubins and Friedman, 1948; Berthier et al., 1988)—same feel, but no hedonic value. Additionally, meditation has been shown to affect specifically the unpleasantness ratings of pain, but rarely other aspects (Mills and Farrow, 1981; Kabat-Zinn et al., 1985; Morone et al., 2008; Perlman et al., 2010). Here, being trained in these spiritual techniques decreases an experience's likelihood of activating a specific subroutine. If so, phenomenal experiences can be *decoupled* from the evaluative aspects they usually carry. This puts the centrality of Bewusstseinsethik under question: Experiences do not necessarily have specific values.

## 4. Conclusion: the pruned project of bewusstseinskultur

Let me reiterate how these four challenges may limit Bewusstseinskultur: In order to build a Bewusstseinskultur, the majority of society must get to an agreement about which conscious experiences we want to promote, which to prohibit, and how we want to implement such recommendations. Thus, (i) there should be some specific value x generally associated with any specific type of experience y, (ii) the fact that y-experiences have an x-value should be knowable before one has had the y-experience, (iii) there should be specific causes that bring about such y-experience, and (iv) these causes for y-experience should be controllable. Why? Because (i) ensures that Bewusstseinskultur is a society-wide project, not merely a personal one – for only if there is no variation in an experience's value among individuals can we agree which types of experiences to promote or prohibit; if one loves and another hates cauliflowers, we cannot get an agreement on whether cauliflower-experiences in general should be promoted or prohibited. Condition (ii) allows rationality to have some role—for only if we can know what value an experience will have can we deliberate on whether we want to have it or not. Conditions (iii) and (iv) allow for recommending specific action plans—for only if there are specific causes for experiences can we control that prohibited experiences are not felt, simply by controlling their causes. Parfit challenges (i) and Paul (ii), while the challenge from non-functional views of consciousness targets (iii). And, affecting (iv), if the functions of consciousness are tightly concentrated in the brain, then this limits the effectiveness of societal interventions because we hardly want to mandate brain surgery to exclude all possible internal causes of experiences—if this is at all plausible in a highly interconnected nexus of causal chains like the brain.

Nothing I have said negates Bewusstseinskultur in principle. Instead, it illustrates how important current philosophical developments and empirical findings about the functional underpinnings of experience are for this project. However, it does set some limits: If some types of experiences are associated with some value to a statistically significant degree—hunger, fear, panic, etc.—these must take precedent for a Bewusstseinskultur (see Metzinger, 2017b). In such cases, strong regulations of such experience's causes are justified. For experiences that are not significantly associated with a specific value, hardly any general recommendations can be made. Instead, we should improve the circumstances of individuals to make their own autonomous decisions and accept the desire for revelatory phenomenal transformations. But we cannot make decision for them. Bewusstseinskultur must then be *anti-authoritarian*. But because the transformations triggered by some experiences are unpredictable, Bewusstseinskultur must also focus on providing coping strategies once transformations went awry. Think of trip guides: Even if we allow people to have LSD trips, we still want to provide them with the best care if the trip turns south (see also Metzinger, 2006b). Then, Bewusstseinskultur is rather caring then commanding, rather liberal than paternalistic. I believe that even this pruned project is worth being pursued, and we are in desperate need of it.

## Author contributions

The author confirms being the sole contributor of this work and approved it for publication.

### Conflict of interest statement

The author declares that the research was conducted in the absence of any commercial or financial relationships that could be construed as a potential conflict of interest.

## Footnotes

^1^Get used to it: Zeitgeist, Doppelgänger, Bildungsroman, Götterdämmerung, Realpolitik, Weltanschauung, Schadenfreude, Wunderkind, Gretchenfrage—impactful neologisms have to be German!

^2^Some practical examples include Metzinger (2006a): How should we deal with psychiatric disorders? How should we treat animals which are unarguably sentient? Which experiences can we impose on them, which one's should we avoid? How should we treat those non-humans where we cannot exclude the possibility that they are sentient (robots, computer-programs, fishes, insects, etc.)? How should we structure our media landscape, which imposes experiences on us and shape our experience of the world and others? How should we teach and which experiences do we want to further? How should a rational drug policy look like that takes into account the undeniable desire of some for psychedelic experiences? How should our last living moments feel like? What costs on society are justifiable to enable hospitals to give us this specific, maximally desired death experience?

^3^Just in line with Marx's creed: “Die Philosophen haben die Welt nur verschieden interpretirt [sic]; es kommt aber darauf an, sie zu verändern.” (Marx, 1845/1998, §11)—Philosophers have only interpreted the world in different ways. What is crucial, however, is to change it.

^4^An exception are chapters 8 and 9 of *The Ego Tunnel* (Metzinger, 2009a), where Bewusstseinskultur is presented under the heading of the “Third Revolution.”

^5^This is akin to what happens in anesthetic awareness (Topulos et al., 1993; Schwender et al., 1998; Ghoneim, 2000; Alkire and Miller, 2005).

^6^There is a notable difference between Sellars' and Metzinger's stance toward the manifest image. According to Sellars, our scientific image neither need to replace nor alter our manifest image. He agrees that there appears to be a “clash of the images” and “conflicting claims” (Sellars, 1991, p. 25). But, “the conceptual framework which I am calling the manifest image is, in an appropriate sense, itself a scientific image” (Sellars, 1991, p. 7) and the goal is to see how “*the manifest and scientific images could merge without clash in the synoptic view* [a kind of fusion view where we simultaneously hold both images]” (Sellars, 1991, p. 34, emphasis in the original). Metzinger, in contrast, holds (often implicitly) that there is a true clash between the scientific and manifest image. The clash not necessarily from the metaphysics of the scientific image, but from shifts in preference in the community between often vulgar interpretations of this image. These shifts may pose a danger and come with emotional costs. As an example, consider this passage on will: “The unsettling point about modern philosophy of mind and the cognitive neuroscience of will, already apparent even at this early stage, is that a final theory may contradict the way we have been subjectively experiencing ourselves for millennia. […] There will be a conflict between the biological reality tunnel in our heads and the neuroscientific image of humankind, and many people sense that this image might present a danger to our mental health” (Metzinger, 2009a, p. 127). In many debates, Metzinger pleads for taking science seriously – his approach is often reductionistic and in parts eliminiativistic, rather than stereoscopic. Our best anthropology is not founded in the implicit normativity of our society and our language games, but in empirical adequacy and theoretical elegance (Metzinger, 2012): “The formal beauty of theoretical simplicity is deadly and creative at the same time. It destroys superfluous assumptions whose falsity we just cannot bring ourselves to believe, whereas truly elegant explanations always give birth to an entirely new way of looking at the world. What I would really like to know is this: Can the fundamental insight the destructive, creative virtue of simplicity be transposed from the realm of scientific explanation into culture or onto the level of conscious experience?” If there is intuitive resistance that keeps us from letting go of ideas of the manifest image, we can explain this the manifest image's intuitive force scientifically—and thereby explain the claims away by explaining why we hold them despite being unfounded (Metzinger, 2004, p. 592). He tries to show the beauty in this scientific approach, a potential to grow if we adopt it thoroughly—something which he adopted from the Churchlands: “If we […] began to train our native mechanisms of introspection with the help of the new and much more fine grained conceptual distinctions offered by neuroscience, then we would also discover much more, we would enrich our inner lives by becoming materialists” (Metzinger, 2009a, p. 53). So he accepts the charge that he is a scientific realist– in the sense that science, not our intuitions or practices, tells us what is real (Metzinger, 2006d, p. 1). Now, how brutal is the clash between Sellars and Metzinger? It might be calmer than we expect: For Sellars, the tenacity of the manifest image is based in the normativity engrained in our use of language. Components of the scientific image can make it into the manifest image, but often under hard labor. Metzinger, as I read him, simply wants to reduce that labor. We, as a community of speakers, can change the implicit norms of our language games in order to make it easier for our best scientific theories to be reflected in the way we speak about ourselves—and therefore, how we view ourselves. In the following, I will base my essay on this interpretation of Metzinger's approach and leave Sellarsian themes and exegeses aside.

^7^To take-up the contrary intuition that the normative value of an experience relies on linguistic practice, one could admit that our linguistic practices largely follow, and therefore reflect the normative force already pre-linguistically present in the experiences themselves.

^8^These are not uncommon descriptions in lexico-gustatory synaesthesia (Simner and Ward, 2006; Simner, 2007; Simner and Haywood, 2009; Colizoli et al., 2013).

^9^Non-functionalism vis-à-vis phenomenality is often associated with Block (1996, 1997, 2005, 2007a,b, 2013).

^10^However, Lewis (1980) might offer a way out—but one that, arguably, waters functionalism down.

^11^This is a reformulation of Parfit's idea in Metzingerian terms.
